# NRE: a tool for exploring neutral loci in the human genome

**DOI:** 10.1186/1471-2105-13-301

**Published:** 2012-11-14

**Authors:** Leonardo Arbiza, Elaine Zhong, Alon Keinan

**Affiliations:** 1 Department of Biological Statistics and Computational Biology, Cornell University, 102 Weill Hall, Ithaca 14853, USA

## Abstract

**Background:**

Analyzing regions of the genome where genetic variation is free from the confounding effects of natural selection is essential for many population genetic studies. Several recent studies in humans have stressed the large effect of natural selection at linked neutral sites and have shown that the choice of putatively neutral regions can have a marked effect on estimates of demographic history.

**Results:**

NRE (Neutral Region Explorer) provides a mechanism for the easy extraction and analysis of nearly neutral regions from the human genome. It can combine many genomic filters, including filters for selection, recombination rate, genetic distance to the nearest gene, percent overlap with annotated regions, and user-provided loci. The program implements a two-step filtering process for greater versatility, allowing users to compile a basic set of neutrality criteria, explore their effect, and use this knowledge to refine filtering. Results can be instantly downloaded in standard formats, along with summary and ranking statistics, or exported to genome browsers such as those from the 1000 Genomes and UCSC. The applicability and value of NRE are demonstrated through an example in the estimation of the ratio of chromosome X-to-autosomal effective population size using different strategies for the selection of neutral regions.

**Conclusions:**

The combined features of NRE make possible the sort of flexible, rigorous mining and analysis of neutral loci increasingly demanded by population genetic studies. NRE is available at http://nre.cb.bscb.cornell.edu.

## Background

Analyzing regions of the genome that are not affected by natural selection is essential for many population genetic studies. While the attention of most large databases has focused on the annotation of functional or genic regions, neutral variants provide a means of understanding a population’s history and a device for gauging the effects of natural selection (e.g. [1]). Several recent studies in humans have shown the large effect of natural selection at linked neutral sites [2-4] and that the choice of putatively neutral regions can have a marked effect on population genetic estimates [5,6]. This effect, attributed to hitchhiking or background selection, is a function of the recombination rate and strength of selection at linked sites [7]. Hence, obtaining neutral regions requires rigorous data filtering to exclude functional elements, error-prone regions, as well as the effects of selection at linked neutral sites.

We have built NRE (Neutral Region Explorer), a database-driven tool that allows experimental and computational biologists to mine non-genic, non-functional regions of the human genome for analysis or targeted sequencing. It is designed to isolate loci that are as neutral as possible by filtering for a variety of criteria including distance from genes, overlap with different types of genomic elements, region size, nucleotide diversity, and the action of selection. Data is presented through a flexible and easy to use interface, allowing users to explore the effects of parameters and automatically sort or rank results, separately or simultaneously, by chosen criteria. Results can be instantly exported in standard formats or visualized along with metadata statistics.

We demonstrate the utility of the data and approach implemented in NRE in contrasting diversity between chromosome X and the autosomes, confirming sex-biased processes during human evolution [5,8-10]. Additionally, this scheme has been used to design targeted next-generation sequencing experiments in a large cohort (in submission).

## Implementation

The approach of NRE is to first exclude undesired (i.e. putatively non-neutral or difficult to sequence) genomic elements, then rank the remaining regions for neutrality and data quality based on a set of estimated parameters. For the first of these steps, the program intersects “hard” filters specified by BED files, of which seven are provided as detailed below to restrict to loci that are, e.g., non-genic, non-conserved, non-repetitive. It then calculates for each region the distance to the nearest gene (physical and genetic distance to the nearest RefSeq transcript [11]), recombination rate (cM/Mb), nucleotide diversity (π), the predicted effect of background selection [2], and percent overlap with other undesired genomic elements (“soft” filters, of which three are provided). The user can upload additional filters of any type in the form of BED files and also has the option of filtering a priori by distance to the nearest gene, recombination rate, and chromosome(s), as well as by the minimum or maximum desired length of resulting regions.

The rationale of these “hard” filters is that duplicated or repetitive regions can pose technical sequencing and assembly challenges which can lead to decreased data quality, while genic regions and conserved elements are more often the target of natural selection. For the estimated parameters, strong reductions in diversity can be indicative of natural selection, while regions far from loci under selection or with high recombination rates are less likely to be affected by the action of selection on linked sites. Finally, the minimum region size filter allows eliminating short runs of contiguous bases, some of which may be as small as a single base, depending on the overlap of selected genomic filters. In combination, these filters exclude loci that are small, are affected by selection, or are in error-prone regions.

In the second step, users can view statistics on the resulting data set and choose to further filter or sort, individually or in combination, by any of the parameters. Sort direction, multiple sort order, filtering maxima and minima, minimum separation among loci, and the number of results to return can be specified by a simple form of text input and check boxes. This allows the user to flexibly choose the best set of regions for their specific purpose. For example, users seeking neutral regions for a targeted sequencing experiment are likely to require different optimal sequence characteristics —e.g. region size, sequence properties, and number of regions— than users intending to aggregate genomic patterns of variation for large-scale population genomic studies.

The resulting regions can be inspected in NRE, downloaded in tabular format, or exported with annotations of estimated parameters to the UCSC genome browser [12] and 1000 Genomes Browser, where further analysis or the extraction of sequences, alignments, and genetic variation data are available.

### Resources

NRE integrates several sources of current data from a variety of public resources. Genetic variation data is currently obtained from the low coverage sequencing pilot of the 1000 Genomes Project Consortium [13] based on the hg18 build. We expect to upgrade to the hg19 build and more recent phases of the 1000 Genome data as they are made public. Two SNP call sets are provided. The merged set is a consolidation of call sets from the Sanger Institute, Broad Institute, and University of Michigan, and constitutes SNP calls based on a larger sample set adequate when comparing across autosomal loci. The Sanger Institute call set was produced with SNP calling software accounting for male hemizygosity on the X chromosome [13] and is provided on NRE only for female individuals, resulting in a more uniform ascertainment for comparisons of variation in chromosome X and the autosomes [10]. The last ~50 Mb of the X chromosome are currently excluded since 1000 Genomes pilot data was not available beyond position 100 Mb.

Recombination rates are included as sex-averaged recombination rates from the HapMap II recombination map [14], pedigree based Decode estimates [15], or the recent admixture based African American map from Hinch et al. [16]. HapMap II recombination rates for chromosome X were mapped over from the hg17 build provided in HapMap using Galaxy’s LiftOver tool, and scaled by 2/3 to account for the effect of no recombination in males [17].

The effect of background selection/hitchhiking is the mean expected fraction of neutral diversity (B) per base as obtained by McVicker et al. [2] for a collection of windows of varying size along the human genome. To estimate the background selection coefficient for a region produced by NRE, B estimates for all windows from McVicker et al. [2] that overlap the region are averaged while weighting by the fraction of bases from the region overlapped by each window.

Finally, genome regions denoted in BED files can be uploaded by the user or selected from those provided. NRE uses the UCSC provided software featureBits [12] and also BEDTools [18] to merge and calculate overlap among tracks. Seven genomic “hard” filters and three “soft” filters obtained from the UCSC genome browser are readily available to NRE users. Gene annotations are obtained from the set of UCSC known genes [12,19], Reference Sequence collection [11], and Gene bounds determined by the full RefSeq gene transcripts [12,20]. These are used to exclude regions as well as to calculate physical and genetic distance to the nearest gene. A filter for conserved elements in placental mammals, including noncoding regions, is also provided (28-Way Most Conserved Placental [21-25]). Users seeking to exclude other types of elements can do so by uploading alternative or complementary filters as BED files. Various filters for repetitive and duplicated regions are also provided: Segmental Duplications [26,27], Copy Number Variants [28,29], Self Chain [22,24,25] (excluded gaps longer than 1 kb, in order to expose a 90 Mb region on the X chromosome), Simple Repeats [30], and Repeat Masker v3.2.7 [21-25]. Note that while Repeat Masker is provided in full as a soft filter, the hard filter option provides a reduced version that includes only those retrotransposons with divergence less than 20% from the consensus sequence.

### Calculations

Nucleotide diversity (π) is estimated as the average number of pairwise differences per nucleotide in each region across individuals, using SNPs from either CEU (from Utah with Northern and Western European ancestry), YRI (Yoruba from Ibadan, Nigeria), or the combined set from East Asian individuals (CHB+JPT) in the pilot phase of the 1000 Genomes Project [13]. Average recombination for a region is taken to be the average of all rates for markers contained in the region and the weighted average of the nearest flanking markers. Distance to the nearest gene is calculated as the distance from the first or last base in the region to the nearest edge of a RefSeq annotated transcript or user uploaded definition of genes, using either the HapMap II [14], Decode [15], or Hinch et al. [16] genetic map to estimate genetic distance.

### Filtering, sorting, and selecting independent loci

Both filtering and sorting options are available in the second step to allow refinement of the initial data set. Filtering takes place as in the first step, by soliciting text inputted minima and/or maxima, with the additional options to specify minimum and maximum nucleotide diversity and percent overlaps with the soft filters. Sorting modules were developed in R [31]. To sort on a single parameter the user selects the corresponding checkbox and either decreasing or increasing order. To sort on multiple parameters simultaneously, the user selects the corresponding checkboxes, inputs the order of priority of each sorting parameter, and specifies a number of bins. Multiple sorting is executed by sorting on the first variable, binning the results, sorting on the second variable within each bin, and so on. As such the number of bins corresponds to the smoothness of multiple sorting: larger number of bins results in finer subsequent sorting.

In a separate box, the user may also choose to retain only a subset of the filtered loci which are separated by a minimum physical or genetic distance from each other. Note that a simple filter based on a measure of separation among neighboring loci can prove largely suboptimal, overshooting the desired property by unnecessarily removing long stretches of loci that are linearly spaced at small intervals. While the optimal solution to this problem prohibitively grows in complexity with the granularity of filters and number of loci, we have implemented a simple heuristic to provide a reasonable balance between computation time, the number of loci retained, and their cumulative coverage in the final set produced by this filter. The algorithm works by iterating over all available loci, moving from the largest to the smallest, and keeping a locus only if it meets the criterion of minimum distance from all loci that were already kept in previous iterations. This filter is applied, as a last step, on the set of loci left after the application of all other filters selected. Finally, the user has the option to obtain only a specified number of top results from the filtered and/or sorted set.

## Results and discussion

To demonstrate the applicability and value of NRE, we estimated the ratio of chromosome X-to-autosomal effective population size (Nx/Na) using different strategies for the selection of neutral regions. Briefly, this ratio has received considerable recent attention [5,8], and in a panmictic population of constant size, with equal sex ratios and reproductive success, it is expected to be 0.75. Deviations from this expectation can result from several factors including, but not limited to, sex-biased demographic processes, changes in population size, natural selection, and differences in mutation rates between the sexes or between chromosome X and the autosomes [8,32,33].

Using NRE, we used the initial hard filtering step to obtain a set of non-genic, non-conserved, non-repetitive regions. Non-genic regions were chosen by selecting the UCSC Known Genes, Gene Bounds, and Spliced ESTs filters, while Segmental Duplications, and Self Chain were used to eliminate regions with duplications. The 28-way Most Conserved Placental Mammal elements and Simple Repeats filters were chosen as soft filters, selecting the maximum tolerated overlap to 0% in the second filtering step (equivalent to a hard filter). One additional hard filter, an outgroup mask containing regions of poor synteny with macaque [22,24,25], was uploaded to the server. For the purpose of obtaining more robust per-region divergence estimates, only regions at least 1000 bases long were included in the analysis, which was easily accomplished by inputting a minimum region length of 1000 bp. Diversity estimates were automatically calculated by the server for both CEU and YRI populations using SNPs produced by the Sanger Institute in female subjects. We estimated divergence with the primate outgroup as the fraction of differences between the human reference sequence and outgroup genome corrected for recurrent mutation by the Jukes Cantor method [34].

**Figure 1:**
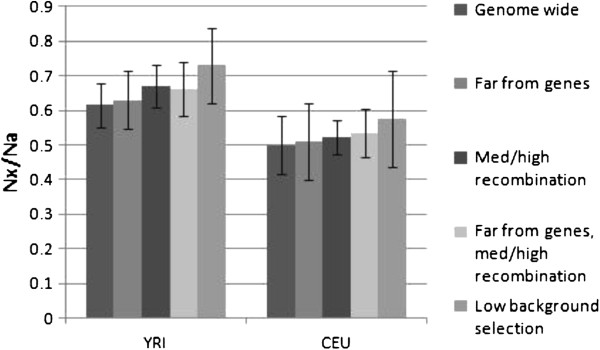
**Effect of neutral region choice in estimating the X-to-autosome effective population size ratio.** Nx/Na (y-axis) is estimated by means of the X-to-autosome ratio of nucleotide diversity (π) normalized by human-macaque divergence to correct for variation in mutation rates. Bars are as described in text. Error bars are standard errors estimated by bootstrapping 10,000 data sets. YRI, Yoruba (Ibadan, Nigeria); CEU, CEPH (from Utah with Northern and Western European ancestry).

We estimated Nx/Na in the resulting dataset and in four subsets resulting from additional filtering in NRE (Figure 1). The four subsets are: (i) regions further than 100 kb from autosomal genes, or 50 kb from X-linked genes [35] (obtained in separate queries by inputting “1-22” and “X” respectively in the “Chromosomes” field), (ii) regions of medium to high recombination rate (r ≥ 0.9 cM/Mb) [35], (iii) the combination of criteria (i) and (ii), and (iv) low predicted levels of background selection (fraction of neutral diversity ≥ 0.75). Visibly, when averaging over all regions, the X-to-autosome ratio is lower in the CEU sample than in YRI and is consistent with previous results [8,10]. The ratio, as well as the individual estimates in both chromosome X and the autosomes (see Additional file 1: Figure S1), grow incrementally in both populations as we apply one or more of the additional stringency filters, and suggest stronger diversity reducing selection at linked sites on the X-chromosome relative to the autosomes, consistent with previous results based on genetic distance from the nearest gene [5,10]. This result is not affected by the use of an alternative outgroup (Additional file 1: Figure S2). Interestingly, the relative ratio, comparing Nx/Na between CEU and YRI populations, remains at ~0.8 across the different filtering schemes (Additional file 1: Figure S3). This suggests as did Gottipati et al. [10] that while Nx/Na within populations shows a clear influence of selection, the difference in the ratio between populations is likely due to demographic rather than selective effects.

Table 1 shows total base counts after each filtering and masking step employed in the example X-vs.-autosomes analysis detailed above. Indicative of the scarcity of truly neutral loci, the final regions set constitutes a small percentage of the genome. Nonetheless it comprises a large number of loci and is conducive to well-powered analysis of both the autosomes and chromosome X (Table 2). NRE can narrow down the data set in a stepwise fashion, increasing stringency with each additional filter, or it can apply all filters simultaneously in the first step and immediately return the most stringent set of loci. While the use of provided filters and conservative default parameters in NRE will provide a quite stringent set of nearly neutral regions, NRE does not purport to decide for the user the optimal balance of stringency and power. Instead, it gives the user the ability to define criteria, explore the tradeoff between stringency and power, and choose those that are optimal according to the requirements of their particular experimental design or analysis.

**Table 1:** Megabases remaining after each filtering and masking step

	***Hard filters***	***Length >1kbp***	***0*%*Simple Repeats***	***Far (bp) from genes***	***Med/high recombination***	***Far (bp) from genes, med/high recombination***	***High BG selection coefficient***
A	1921.62 (65.0%)	676.70 (35.2%)	522.88 (77.3%)	267.28 (51.1%)	120.17 (23.0%)	54.59 (10.4%)	395.32 (75.6%)
X	97.99 (63.3%)	43.30 (44.2%)	20.56 (47.5%)	14.63 (71.1%)	3.08 (15.0%)	2.15 (10.5%)	10.31 (50.2%)

The first three filters, starting with the leftmost column were sequentially applied, resulting in the “genome-wide” set on which all additional analyses are based for both the X-chromosome (X) and the autosomes (A). Subsequent filters are all subsets of this set. Indicated percentages are out of the previous filtering step, i.e. previous column and the third column for all following columns.

**Table 2:** Genome-wide macaque-normalized diversity estimates and ratios of chromosome X to autosomes

**Pop**	**#Mb X**	**Normalized X diversity**	**#Mb A**	**Normalized A diversity**	**Normalized X/A diversity**
CEU	20.6	0.00797 (0.0015)	522.9	0.01596 (0.0003)	0.4992 (0.083)
YRI		0.01245 (0.0008)		0.02023 (0.0001)	0.6154 (0.064)
CEU/YRI		0.63980 (0.0897)		0.78890 (0.0162)	0.8113 (0.115)

Estimates for each region (and standard errors) together with the total number of bases analyzed after filtering (Mb). Note that the genome-wide data summarized in this table correspond to the leftmost bars of Figure 1 and Additional file 1: Figure S3 prior to the application of the more stringent set of filters for neutrality which have a large effect on results.

The two-step scheme of NRE facilitates such comparisons of neutral data sets of increasing stringency and their effect on measures of interest. Demonstrably, NRE can be readily employed to select regions for human demographic analysis of the kind now rising in frequency and to disentangle the effect of demographic history from that of natural selection.

## Conclusion

NRE is a unique tool that offers a service of increasing demand for genomic scientists. As more studies are devoted to elucidating human evolutionary history, there will be an increasing and more acute demand for tools for analyzing neutral regions. NRE provides an easy to use platform for mining and customizing rigorously defined neutral regions and should prove useful for large scale resequencing design, demographic modeling, and studies of natural selection. It has the advantage of flexibility and ease of use, of coordinating with existing genomic resources, and of being a one-stop hub for an assortment of current, useful data. Importantly, NRE shares simple data processing capabilities with hubs of genomic information or collections of flexible tools such as the UCSC genome browser [12] or Galaxy [36]. However, NRE specifically addresses the task of mining for neutral regions in the human genome through an otherwise laborious integration of different filters, data sources, and data types: genic and conserved regions, data quality filters, genetic maps, genotypes from different human populations, the ability to obtain estimates of diversity and the effect of selection at linked sites, together with the considerations required for accurate comparisons of estimates between autosomal and sex-linked loci. NRE thus addresses a current gap that is not easily covered by existing resources, providing a reproducible strategy, that is well integrated with and thus complimentary to other existing and familiar tools available to the genomics community.

## Availability and requirements

NRE is available at http://nre.cb.bscb.cornell.edu. It is platform independent and supported on current versions of web browsers that support JavaScript and CSS. It is available for use at no charge and without a login requirement or restrictions on usage.

## Competing interests

The authors declare that they have no competing interests.

## Authors’ contributions

AK conceived and designed the project. LA and AK designed the web server and algorithms. LA and EZ implemented the algorithms and performed the analyses. LA, EZ, and AK wrote the paper. All authors read and approved the final manuscript.

## Supplementary Material

Additional file 1 **Figure S1.** Diversity estimates normalized by human-macaque divergence (π/D) presented in Figure 1 are shown independently for the X-chromosome (X) and the autosomes (A). Error bars are standard errors estimated by bootstrapping 10,000 data sets. **Additional file 1: Figure S2.** Same as main text Figure 1, except for the use of orangutan as outgroup. **Additional file 1: Figure S3.** Relative ratio, comparing Nx/Na among European (CEU) and African (YRI) populations. Error bars are standard errors estimated by bootstrapping 10,000 data sets. Click here for file
