# Supplementary material for: NRE: a tool for exploring neutral loci in the human genome
Source: BMC Bioinformatics. 2012 Nov 14;13:301. doi: 10.1186/1471-2105-13-301 (PMC3543337; doi:10.1186/1471-2105-13-301)
Supplement: Additional file 1 — Figure S1. Diversity estimates normalized by human-macaque divergence (π/D) presented in Figure 1 are shown independently for the X-chromosome (X) and the autosomes (A). Error bars are standard errors estimated by bootstrapping 10,000 data sets. Additional file 1: Figure S2. Same as main text Figure 1, except for the use of orangutan as outgroup. Additional file 1: Figure S3. Relative ratio, comparing Nx/Na among European (CEU) and African (YRI) populations. Error bars are standard errors estimated by bootstrapping 10,000 data sets. [file 1471-2105-13-301-S1.docx]

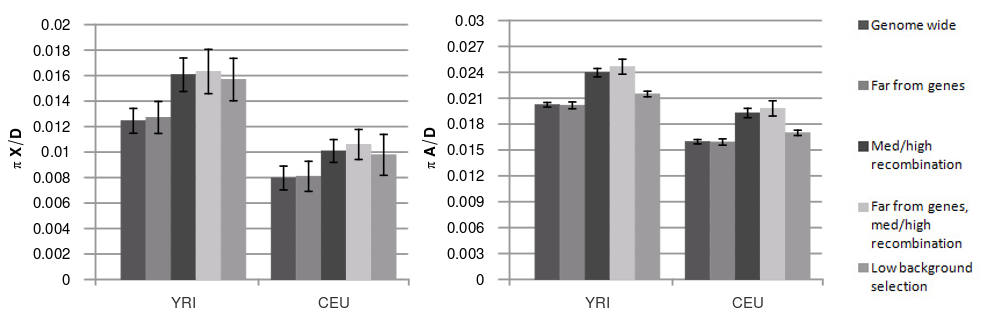


**Figure S1:** Diversity estimates normalized by human-macaque divergence (π/D) presented in Figure 1 are shown independently for the X-chromosome (X) and the autosomes (A). Error bars are standard errors estimated by bootstrapping 10,000 data sets.

**Figure S2:** Same as main text Figure 1, except for the use of orangutan as outgroup.


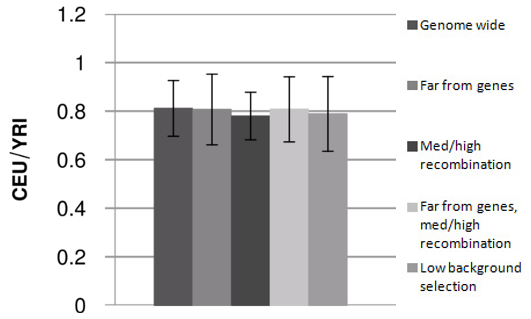


**Figure S3:** Relative ratio, comparing Nx/Na among European (CEU) and African (YRI) populations. Error bars are standard errors estimated by bootstrapping 10,000 data sets.
